# Bazedoxifene and Beyond: Identifying This SERM’s Targets and Deciphering Its Molecular Mechanisms

**DOI:** 10.1155/adpp/3028716

**Published:** 2026-08-04

**Authors:** Juliette Bherer, Alisson Clemenceau, Caroline Diorio, Francine Durocher

**Affiliations:** ^1^ Department of Molecular Medicine, Faculty of Medicine, Université Laval, Quebec, Quebec, Canada, ulaval.ca; ^2^ Endocrinology and Nephrology Research Program, CHU de Québec-Université Laval Research Center, Quebec, Quebec, Canada; ^3^ Cancer Research Centre, Université Laval, Quebec, Quebec, Canada, ulaval.ca; ^4^ Department of Internal Medicine, Section of Endocrinology and Metabolism, Yale School of Medicine, New Haven, Connecticut, USA, yale.edu; ^5^ Department of Social and Preventive Medicine, Faculty of Medicine, Université Laval, Quebec, Quebec, Canada, ulaval.ca; ^6^ Oncology Research Program, CHU de Québec-Université Laval Research Center, Quebec, Quebec, Canada; ^7^ Center for Breast Diseases, Saint-Sacrement Hospital, Quebec, Quebec, Canada

**Keywords:** bazedoxifene, cannabinoid receptor, drug repurposing, ferroptosis, glycoprotein 130, selective estrogen receptor modulator

## Abstract

Bazedoxifene (BZA), a third‐generation selective estrogen receptor modulator (SERM) approved for osteoporosis treatment, is attracting attention for drug repurposing, especially in cancer research. While designed primarily for estrogen receptors, BZA exhibits in vitro off‐target interactions that may explain novel therapeutic benefits or account for potential side effects. This review comprehensively examines both established and newly identified targets of BZA beyond estrogen receptors. To gather relevant data, we reviewed over 1500 articles selecting key studies proposing insights into BZA targets and mechanisms. This review details BZA’s roles in estrogen receptor signaling and its interactions with glycoprotein 130, elaborating on vital components of each signaling pathway. It also compiles its potential interactions with cannabinoid receptors, as well as BZA targets related to biotransformation, absorption/transport, DNA cycle, Sars‐CoV‐2, ferroptosis, ROS production, and cholesterol biosynthesis. For each target, we discuss evidence from computational studies, direct interaction assay, and functional testing. This work provides the most complete overview of BZA’s molecular mechanisms to date and suggests new avenues for future research and potential applications beyond osteoporosis.

## 1. Introduction

Bazedoxifene (BZA) is a third‐generation selective estrogen receptor modulator (SERM). This indole‐based compound binds estrogen receptors (ERs), acting as an agonist or antagonist in a tissue‐specific manner [1]. During menopause, declining estrogen levels increase the risk of osteoporotic fractures, making selective activation of ER in bone tissue therapeutically relevant [2, 3]. Therefore, BZA was approved in 2009 by the European Medicines Agency and in 2010 by Japan’s Pharmaceuticals and Medical Devices Agency for the treatment of osteoporosis in postmenopausal women at high risk of fractures [4, 5].

Recently, BZA has been the subject of drug repurposing studies and notably for cancer treatment [6, 7]. Drug repurposing investigates the potential of already approved drugs for new indications, offering a faster and more cost‐effective alternative to developing novel compounds, which require target identification, preclinical testing, and clinical trials to establish efficacy and safety [8, 9]. Although BZA was designed to target ER, off‐target interactions have also been explored [10, 11]. Such mechanisms may contribute to side effects or therapeutic effects, as seen with other SERMs, highlighting the importance of characterizing BZA’s molecular targets beyond ERs to evaluate its potential new applications [12].

Thus, this review aims to examine the established interactors and molecular mechanisms of BZA, while highlighting emerging interactors that may represent novel therapeutic targets or off‐target effects. To evaluate the strength of evidence, we detail for each interaction: in silico testing, proof of physical interactions through in vitro testing, and functional assays (in vitro and/or in vivo). Where possible, we also discussed whether these mechanisms are shared with other SERMs such as tamoxifen (TAM) and raloxifene (RAL).

To have a scope of related BZA literature, MEDLINE and Embase were investigated for in silico, in vitro, and in vivo studies using the keywords: BZA or conbriza or tse‐424 or tse424 or viviant or way‐140424 or way140424. Over 1500 abstracts were screened. If BZA was only mentioned in the introduction or the discussion, the article was excluded. Published conference abstracts, book chapters, and reviews were also excluded, as methodological information is often incomplete. Hence, this review provides a comprehensive summary of known or proposed BZA targets up to August 12, 2025. Revisiting these studies in the context of current knowledge highlights new research questions for further characterization of BZA’s molecular roles and potential applications.

i. This review provides a distinct perspective on BZA by adopting a target‐centric, rather than disease‐centric, framework. Such an approach enables a systematic evaluation of reported protein interactions, organized according to the strength of available evidence, while directly linking these mechanisms to their potential clinical relevance. By structuring the literature in this way, the review aims to better support future research directions and drug repurposing strategies.
ii. In addition, this work contextualizes BZA’s molecular targets within the broader class of SERM by explicitly identifying which interactions are shared with two other main SERMs and which may represent BZA‐specific properties. This distinction provides a clearer understanding of SERM effects *versus* potentially unique pharmacological features.
iii. Finally, this review offers the first dedicated synthesis of emerging evidence connecting BZA to reactive oxygen species (ROS) modulation and ferroptosis‐related pathways, areas that have not been comprehensively addressed in previous reviews.

## 2. Brief History

Hormone replacement therapy was long used to alleviate menopausal symptoms, including osteoporotic fractures [13]. While effective, hormone replacement therapy also increases the frequency of strokes and the risk of hormone‐dependent cancers [14]. To avoid such complications, another therapeutic avenue was explored: molecules known as SERMs. In contrast to hormone replacement therapy, SERMs do not have the steroid structure of estrogen but can still interact with ER [14]. TAM, a first‐generation SERM, treats ER‐positive (ERα+) breast cancer but can induce uterine hyperplasia, thereby increasing the risk of endometrial cancer [15]. RAL, a second‐generation SERM, increases bone mass yet still stimulates uterine cells [16]. This highlighted the need for a SERM that acts as a bone agonist while antagonizing uterine receptors, leading to the introduction of TSE‐424 (now BZA) by Miller et al., also named as 1‐[[4‐[2‐(azepan‐1‐yl)ethoxy]phenyl]methyl]‐2‐(4‐hydroxyphenyl)‐3‐methylindol‐5‐ol, a third‐generation SERM in 2001 [1].

Preclinical studies in ovariectomized (OVX) rats and primates showed that BZA increased bone density without inducing uterine hyperplasia or significant changes in uterine weight [1, 16, 17]. In postmenopausal women with osteoporosis, multicenter Phase 3 trials demonstrated that BZA reduced vertebral and nonvertebral fractures while maintaining a safety profile comparable to placebo, with no significant stimulation of uterine or breast tissue [18–32]. Separate trials in postmenopausal women without osteoporosis confirmed BZA’s efficacy in preventing bone loss, reducing bone remodeling biomarkers, and maintaining a side effect profile similar to control groups [33–35]. Overall, BZA was as effective as RAL in both treating osteoporosis and preventing its onset. Consequently, BZA was approved for use in Europe (2009) and Japan (2010) and is marketed as Conbriza and Viviant, respectively [4, 5]. In the United States, it is available only in combination with conjugated estrogens (CEs) under the brand name Duavee [36].

## 3. Pharmacokinetics and Clinically Relevant Concentrations

In healthy postmenopausal women receiving oral BZA at 20 mg once daily for 30 days, the reported maximum plasma concentration (*C*_max_) is 7.157 ng/mL, corresponding to approximately 15.2 nM [37]. Accordingly, interactions occurring within the nanomolar range are the most consistent with clinically achievable systemic exposure.

In contrast, reported effects occurring at micromolar concentrations, which exceed the estimated maximum plasma level by several orders of magnitude, are unlikely to be systemically relevant under standard dosing conditions. However, several factors may modulate local exposure. BZA is highly lipophilic and exhibits extensive tissue distribution [38, 39]. In a Phase I study, BZA apparent volume of distribution was high (122 ± 70 L/kg; range 16–268 L/kg), consistent with extensive tissue penetration [37, 40]. Preclinical studies further suggest substantial tissue accumulation: In male rats, radiolabeled BZA concentrations were approximately 10‐fold, 18‐fold, and 55‐fold higher in the kidney, lung, and liver, respectively, compared with plasma levels 8 h after oral administration (3 mg/kg) [39]. In addition, modeling approaches predict the distribution of BZA from plasma to multiple organs in humans, including the lung, brain, and heart [41]. While these observations indicate that plasma concentrations may not fully reflect local exposure, the extent to which tissue levels reach pharmacologically active concentrations for nonclassical targets remains uncertain.

To address these limitations, we applied a structured pharmacological relevance framework integrating exposure data (relative to *C*_max_), tissue distribution considerations, and level of evidence determined by a scoring system to distinguish clinically plausible interactions, potentially plausible under specific tissue or exposure conditions, or unlikely to be relevant in humans (Table 1; Supporting Table 1). This approach aims to distinguish mechanistically supported interactions from those that remain speculative based on current data.

**TABLE 1:** Summary of proteins explored in the literature related to BZA along with the corresponding strength of evidence.




*Note:* Blue boxes denote the documented interactions, whereas white boxes indicate the absence of reported evidence. The strength of evidence (strong = green, moderate = yellow, weak = red) was based on the type of experimental evidence, number of supporting studies, presence of conflicting studies, and concentrations used. The criteria for evidence classification are further detailed in Supporting Table 1.

## 4. ERs: Primary BZA Interactors

In 2001, Miller et al. showed that BZA directly binds ERα competing with estradiol (E2) at low nanomolar concentration. Using radiolabeled E2, the concentration of BZA required to displace 50% of ligands was 23 ± 15 nM, later refined to a median inhibitory concentration (IC50) of 8.2 ± 1.1 nM [1, 42]. *In silico* analyses further confirmed high BZA–ERα affinity [43–48]. BZA docking scores across known or putative targets are summarized in Supporting Table 2 and are hypothesis‐generating data to prioritize likely interactions [10, 11, 38, 44–55].

Functionally, in MCF‐7 breast cancer cells, BZA acted as an ERα antagonist, inhibiting estrogen‐responsive gene expression with half‐maximal effect at 3.7 ± 1.6 nM^1^. Consistent with its SERM classification, it shows antiestrogen activity in breast and uterine tissues, while displaying agonist effects in bone, liver, and adipose tissue [16, 56–69]. In addition, BZA (low nM–1 μM) exhibits partial selective estrogen receptor degrader (SERD)–like activity by promoting ERα degradation in breast cancer models, although less effectively and in a context‐dependent manner compared to classical SERD [43, 56, 70, 71].

While several narrative reviews have addressed BZA–ERα interactions from a pathology‐centered perspective [72, 73], the following sections shift the perspective toward a detailed examination of the molecular components of ER signaling in relation to BZA treatment.

### 4.1. ERα Domains and Mutations

**FIGURE 1:**
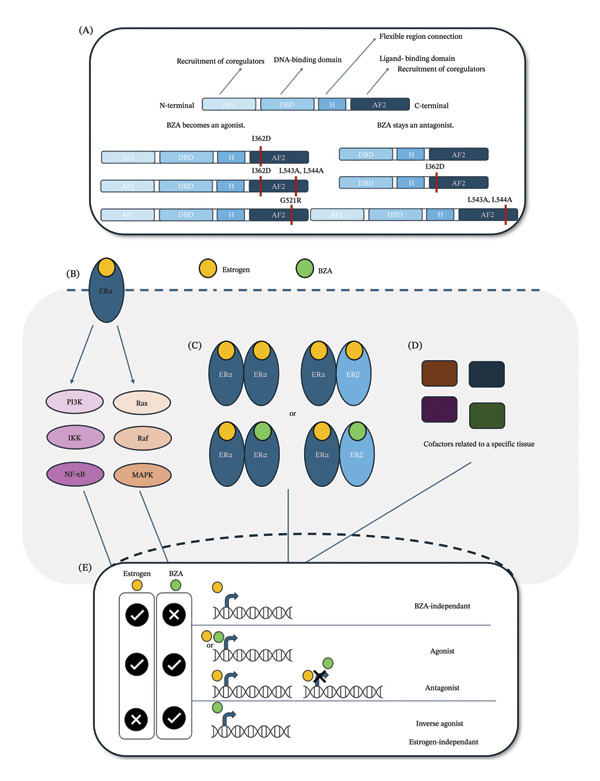
Key unresolved aspects and specific aspects of ER signaling relevant to BZA action: (A) Mutations within the AF2 domain can convert BZA from an antagonist to agonist, an effect prevented by AF1 loss. Red lines indicate the mutation sites; (B) membrane‐associated ER signaling pathways potentially modulated by BZA; (C) ERα homodimerization *versus* heterodimerization, including heteroligand dimers; (D) tissue‐specific interactors that influence whether BZA acts as an antagonist or agonist; (E) distinction between estrogen‐dependent and BZA‐specific gene regulation. Abbreviations: BZA = bazedoxifene, ER = estrogen receptor, AF1 = activation function‐1, DBD = DNA‐binding domain, H = hinge, AF2 = activation function‐2, PI3K= phosphoinositide 3‐kinase, IKK= IκB kinase, NFκB = nuclear factor‐kappa B, RAS = rat sarcoma virus, MAPK = mitogen‐activated protein kinase.

ERα consists of four domains: activation function‐1 (AF1), DNA‐binding domain, hinge region, and activation function‐2 (AF2). AF1 recruits coregulators such as SRC1 and CBP‐1, the DNA‐binding domain binds the estrogen‐responsive element and AF2 mediates ligand binding and coregulator recruitment (Figure 1A) [73].

BZA stabilizes the ERα ligand–binding domain in a manner similar to RAL [74]. Its indole moiety forms a stabilizing hydrogen bond with Glu353, anchoring BZA within the binding pocket [43, 44]. In addition, the azepanyl ring of BZA induces a subtle but significant shift in Helix 12 (found in AF2), a key component for ERα antagonism [43, 70].

In mice expressing AF1‐truncated ERα, BZA treatment (12 μg/day for females and 48 μg/day for males) did not modify bone parameters compared to wild‐type mice, a pattern also observed with E2, inferring that AF1 is required for agonistic activity in bone tissue [57, 75]. In contrast, in HepG2 cancer cells, where BZA (1 μM) acts as an antagonist, AF1 depletion did not impair BZA function, suggesting that AF1 is dispensable for BZA’s antagonistic activity [76].

In comparison, mutations within the AF2 domain may differentially influence BZA activity on ERα. While mutations in Helix 12 (L543A, L544A) did not alter ERα signaling in HepG2 cells, a mutation in the static region (I362D) enhanced ERα activation in response to BZA (100 nM), shifting its activity from antagonism to agonism [76]. Similarly, the AF2 mutation G521R converts BZA (1–1000 nM) into a transcriptional agonist, whereas wild‐type ERα remains unresponsive or repressed [77]. This effect was specific to BZA, as CE did not elicit the same effects with the mutated receptor [77]. Collectively, these studies indicate that some AF2 mutations can convert BZA from an antagonist into an agonist (Figure 1A) [77].

Clinically, most ERα mutations in endocrine‐resistant breast cancer cluster in the C‐terminal AF2 region and are not expected to abolish BZA antagonism [78]. Consistently, BZA retains antiproliferative activity and suppresses ERα target gene expression in TAM‐resistant breast cancer cell lines harboring Y537S, Y537N, or D538G AF2 mutations, with effects comparable to wild‐type models [70, 79, 80]. However, ∼13% of endocrine‐resistant ERα+ breast tumors harbor mutations in Helix 5 of AF2, whose impact on BZA response remains unknown [78].

### 4.2. Localization of ERα: Canonical *Versus* Noncanonical Pathway

The canonical ER pathway is activated when estrogen diffuses into the cell and binds cytoplasmic ERα or ERβ. Upon ligand binding, ER undergoes dimerization and nuclear translocation to regulate gene transcription. Conversely, noncanonical (nongenomic) signaling relies on membrane‐associated ERα or G protein–coupled estrogen receptor (GPER), which activate cytoplasmic pathways such as phosphoinositide 3‐kinase (PI3K) and mitogen‐activated protein kinase (MAPK) (Figure 1B) [81].

In ERα‐negative endometrial cancer cell line engineered to express only membrane‐associated ERα, a high dose of BZA (40 μM) reduced viability and migration via potentially the modulation of focal adhesion kinase and protein kinase B (AKT) phosphorylation, suggesting the involvement of noncanonical pathways, although at supraphysiological concentrations [82]. In MCF‐7 cells, BZA alone (1 μM) did not modify the ERα recruitment to target genes but reduced ERα‐ and extracellular signal‐regulated kinase 2 (ERK2)–mediated transcription in the presence of estrogen, consistent with inhibition of MAPK signaling [83, 84].

In vivo, membrane ERα contributes to tissue‐selective effects: BZA (24 μg/mouse/day) improved bone parameters and altered adiposity in a membrane ERα‐dependent manner [63]. However, noncanonical signaling via GPER does not appear to contribute to the mechanism of action of BZA. Indeed, in vivo GPER knockout did not alter the vascular effects of BZA [85]. Overall, both canonical and noncanonical ER pathways contribute to tissue‐specific responses, although their relative contribution remains incompletely defined.

### 4.3. Dimerization: Heterodimer and Heteroligand Dimer

ERα typically forms homodimers but can heterodimerize with ERβ (Figure 1C) [86]. BZA binds ERβ directly, displacing a radio‐labeled ligand of ERβ at relatively low concentrations (85 ± 59 nM) [1]. However, its affinity is lower for ERβ than for ERα (IC50 > 3x higher) [1]. When benchmarked against clinically achievable exposure, these values suggest that ERβ engagement may fall within a potentially plausible range, although this remains sensitive to the low plasma concentrations and high protein binding characteristic of BZA [5]. Dai et al. further showed that BZA interacts with helices 10 and 11 of ERβ’s ligand‐binding domain [87]. BZA can upregulate both ERβ and ERα in the brain of ischemia‐reperfusion rat models, indicating that interaction with both ER could potentially occur in vivo [88]. Nevertheless, evidence remains preclinical, and no data currently demonstrate ERβ engagement at clinically relevant exposure levels in vivo.

Heteroligand dimers, in which each ERα binds to a different ligand, can regulate gene expression. Cells expressing both a BZA‐preferring ERα and an estrogen‐binding ERα had higher reporter gene expression when treated with both ligands than with either alone, indicating collaborative transcriptional activation (BZA = 10 nM and CE = 100 nM) [77]. Molecular dynamics simulations revealed that heteroligand dimer binding BZA and E2 resulted in an asymmetrical communication throughdistinct helices and residues from those involved in the dimerization of two ERα binding estrogen (homodimer) [89]. These mechanisms remain exploratory; however, heteroligand dimers may have unique regulatory roles and warrant further study, especially when endogenous estrogens are present.

### 4.4. Coregulators

Tissue selectivity of SERMs is largely driven by differential recruitment of coregulators (Figure 1D) [73]. Microsphere‐based multiplex assays showed that BZA (30 nM) markedly suppressed ERα binding to cofactors including CBP‐I, p300‐I, NRIP‐II, MNAR‐II with inhibition ranging from 80 to 90% and moderately reduced ERα binding to SRC cofactors (SRC1, SRC2, and SRC3), with decreases of about 50% compared to CE (10 nM) [42]. In addition, a reduction of SRC protein recruitment to ERα by BZA (100 nM) compared to CE was observed in nuclear extracts from both breast and cervical cancer cell lines [90]. Comparative studies in multiple normal cellular or in vivo healthy tissue models are needed to clarify how coregulator dynamics drive SERM tissue selectivity.

### 4.5. Gene Specificity

BZA alone generally shows minimal transcriptional activity at nanomolar concentrations, with profiles often clustering close to vehicle‐treated conditions (Table 2). Nevertheless, context‐dependent effects are observed, including occasional inverse agonist of E2‐independent modulation of specific gene subsets [42, 91–93].

**TABLE 2:** Key findings of the gene chip and RNAseq of cell lines and tissues treated with BZA alone or in combination with estrogen compounds.

References	Model	Method	BZA	Key findings
[42]	MCF7	Microarray	UNK	BZA alone modulated few genes and showed a transcriptomic profile similar to RAL and vehicle. In combination with CE, BZA altered a subset of CE‐regulated genes but to a lesser extent than RAL or LAS, suggesting weaker antagonism.
[91]	MCF7	Microarray	1 nM or 10 nM	BZA alone minimally affected ER‐responsive genes and clustered with vehicle and RAL. BZA alone also modified the expression of genes that are not changed by estrogen. In combination with CE, BZA antagonized a subset of CE‐induced genes involved in the cell cycle and growth, while leaving others unaffected.
[92]	MCF7	Microarray	100 nM	BZA behaved as an ER antagonist, inverse agonist, partial agonist, or E2‐independent modulator depending on the gene context. BZA alone clustered with vehicle and RAL, while BZA + E2 clustered with RAL + E2, highlighting gene modulation similar to RAL.
[93]	MCF7	RNAseq	5 nM	BZA alone had a minimal transcriptional impact. In combination with E2 or EC10, BZA reduced estrogen‐stimulated gene expression, particularly genes involved in the cell cycle, transcription, and signaling, while also modulating E2‐independent pathways.
[93]	MCF10A	RNAseq	5 nM	BZA showed minimal transcriptional effects, with no clear clustering between treatments, indicating limited estrogen‐dependent or independent activity in nontumorigenic breast cells.
[61]	OVX mouse liver high‐fat diet	RNAseq	3 mg/kg/day	BZA regulated hundreds of genes, both uniquely and shared with E2 or CE. Affected pathways included inflammation, NF‐κB signaling, oxidative stress, and lipid metabolism, indicating strong hepatic activity.
[56]	OVX mouse liver	RNAseq	10 mg/kg/day	BZA induced very limited gene changes and showed transcriptomic similarity to CE and BZA + CE, suggesting modest and overlapping hepatic effects.
[65]	Rat ovary	Microarray	30 mg/kg/day	Short‐term BZA treatment alone significantly altered ovarian gene expression.
[94]	OVX monkey breast	Microarray	20 mg/kg/day	BZA alone and BZA + CEE clustered together and opposed CEE‐driven transcription. BZA suppressed CEE‐induced genes and modestly activated immune‐related genes, supporting breast‐specific antagonistic activity.

Abbreviations: BZA = bazedoxifene, CE = conjugated estrogens, CEE = conjugated equine estrogens, E2 = estradiol, EC10 = estrogen compounds, OVX = ovariectomized, RAL = raloxifene, UNK = unknown.

When combined with ligands, BZA consistently antagonized ligand‐induced transcription, and reduced expression of pathways linked to cell cycle, proliferation, and RNA damage repair (Supporting Table 3) [42, 91–93]. Some genes were uniquely modulated by BZA or by BZA–estrogen cotreatment, suggesting context‐dependent or off‐target effects [42, 91–93]. BZA and RAL showed similar transcriptional patterns [42, 91, 92]. In a nontumoral ER‐negative breast cell line, BZA had limited impact on gene expression, suggesting minimal off‐target effects under low exposure in vitro [93].

In vivo transcriptomic responses are tissue‐ and context‐dependent. Hepatic effects range from minimal gene modulation under basal conditions to broader regulation under metabolic stress, particularly in pathways related to inflammation and lipid metabolism [56, 61]. In contrast, ovarian and breast tissues display distinct and sometimes divergent responses, including estrogen antagonism in breast tissue and context‐specific gene regulation in the ovary [65, 94]. Overall, BZA acts primarily as a ligand‐dependent transcriptional modulator with limited activity in the absence of estrogen signaling.

### 4.6. Clinical Implications

Clinical evidence supports BZA as a tissue‐selective ER modulator acting primarily through canonical ERα signaling, with additional contributions from membrane‐associated pathways. However, mechanistic uncertainties remain that may influence the therapeutic response.

For instance, the roles of ERβ, ER heterodimers, and heteroligand complexes remain poorly defined and may contribute to variability in estrogen signaling contexts. Furthermore, AF2 mutations can alter ERα pharmacology, although most clinically relevant resistance mutations do not abolish BZA antagonism. These findings highlight the potential importance of receptor context and mutation status in modulating the response [95], but current evidence does not yet support routine clinical stratification based on these parameters.

Overall, a more complete understanding of BZA’s transcriptional and noncanonical mechanisms will be important for refining its therapeutic positioning and supporting rational expansion to additional indications.

## 5. Glycoprotein 130: Most Documented Novel Interactor of BZA

**FIGURE 2:**
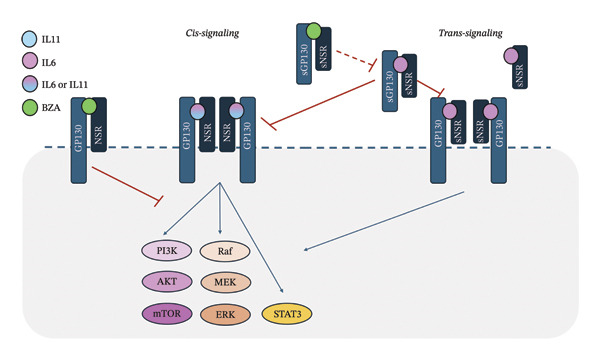
Cis‐ and trans‐signaling of IL6 or IL11 and their possible disruption by BZA. In cis‐signaling, membrane GP130 and NSR bind to IL6 or IL11 to form trimers that dimerize and activate STAT3, PI3K/AKT/mTOR, and MAPK pathways. BZA blocks this activation by inhibiting hexamer formation. In trans‐signaling, the soluble receptor of IL6 (sNSR) and IL6 dimers binds membrane GP130 to trigger the same pathways as cis‐signaling, whereas soluble GP130 (sGP130) limits the availability of IL6‐sNSR dimers, thereby inhibiting IL6 signaling. Replacing IL6 with BZA in trans‐signaling complexes may antagonize both pathway activation and soluble complex–mediated inhibition. The blue arrow represents the activation. The red lines represent the inhibition. The red dashed line represents a potential inhibition. Abbreviations: GP130 = glycoprotein 130, NSR = nonsignaling receptor, sNSR = soluble nonsignaling receptor, sGP130 = soluble GP130, IL6 or 11 = interleukin 6 or 11. This figure was adapted from Martínez‐Pérez et al., [96], and Shi et al., 97, to reflect IL6/IL11 signaling in the context of BZA treatment.

In 2014, glycoprotein 130 (GP130) was identified has a potential BZA’s target through in silico and in vitro analyses [10]. GP130 is a shared signal‐transducing β receptor for IL6 family cytokines including interleukin 6 (IL6), interleukin 11 (IL11), oncostatin M (OSM), and leukemia inhibitory factor (LIF) that enables intracellular signaling by partnering with nonsignaling cytokine α receptors (NSRs) such as interleukin 6 receptor (IL6R) and interleukin 11 receptor (IL11R) [96]. The classical signaling complex of IL6 and IL11 encompasses 2 GP130, 2 NSRs, and 2 ligands, forming a hexamer (Figure 2) [10].

Structural modeling predicted that the indole and azepanyl moieties of BZA bind to hot spots residues with Domain 1 of GP130, mimicking two critical amino acids of IL6 that normally facilitate the binding of the opposite trimer. Through this interaction, BZA disrupts hexamer formation, thereby interfering with the cytokine‐induced signaling [10]. In vitro DARTS assay showed that BZA (10–1000 μM) rescued GP130 from enzymatic digestion, supporting a direct interaction. Functionally, BZA reduced the signal transducer and activator of transcription 3 (STAT3) phosphorylation without affecting total STAT3 levels suppressing constitutive activation in ERα‐negative breast cancer cells and IL6‐induced activation in PANC‐1 cells. Similar effects were observed with RAL [10].

Since these initial findings, more than 20 studies have investigated BZA–GP130 interactions in cancer and noncancer contexts, including inflammatory and cardiovascular diseases [6, 7, 10, 43, 70, 97–123]. Across models, BZA consistently inhibits STAT3 phosphorylation and has also been reported to modulate additional IL6‐associated pathways, including PI3K/AKT/mTOR and MAPK [97, 115, 117–120, 122, 123]. While most studies have centered on IL6‐driven signaling, data suggest broader effects across IL6 family cytokines and raise questions regarding potential roles in trans‐signaling mechanisms. Building on prior reports [97], the following sections broaden this perspective to provide a more integrated view of cytokine networks underlying BZA’s GP130‐mediated actions.

### 5.1. GP130 Cytokine Selectivity

Because BZA may bind GP130, its inhibition is expected to be GP130‐centric rather than cytokine‐specific. Consistent with this, BZA has been reported to inhibit IL11‐driven STAT3 signaling in colon and bone cancer models [114]. In colorectal cancer systems, BZA (15 μM)‐induced apoptosis in IL11‐stimulated cells and reduced tumor growth in HT29 xenografts (3 mg/kg for 14 days), an effect lost upon GP130 depletion [100]. Patient‐derived colon cancer organoids further indicated that BZA efficacy depends on functional GP130, IL6R, or IL11R expressions [100].

In additional studies across multiple cell models (murine B cells, glioblastoma cells, and engineered reporter systems), BZA (0.01–5 μM) inhibited IL11‐dependent viability or signaling [98, 109]. In ERα‐negative breast and gastric cancer cells, BZA (0.003–30 μM) reduced IL11‐induced STAT3 activation without altering total STAT3 levels [98].

In contrast, because LIF and OSM activate GP130 through trimeric receptor complexes that do not require hexamer formation, their signaling is not affected by BZA [96]. Accordingly, BZA did not inhibit LIF‐induced STAT3 phosphorylation in PANC‐1 cells (15–25 μM) or LIF‐stimulated viability in murine B cells expressing human LIFR (0.01–3 μM) [10, 98]. Similarly, OSM‐driven STAT3 activation remained unaltered in colorectal and pancreatic cancer models (5–20 μM) [102, 103].

Together, these data indicate that BZA is not a general GP130 inhibitor in preclinical testing. Instead, its action depends on hexamer inhibition, making it selective for IL6‐ and IL11‐dependent pathways. Assessing the predominant cytokines driving GP130 signaling in disease context will be crucial to guide BZA repurposing, if higher dosage in humans were to be considered. Indeed, most reported effects of BZA related to GP130 occur at micromolar concentrations that exceed the clinically achievable plasma level by several orders of magnitude. Accordingly, GP130/STAT3 modulation by BZA is unlikely to be systemically relevant at the authorized dose. Although isolated, effects are observed at low nanomolar concentrations, but they lack robust in vivo validation.

### 5.2. Soluble Receptors of GP130 Related Pathway: sIL6R and sGP130

IL6 trans‐signaling depends on soluble forms of IL6R and GP130 (sIL6R and sGP130), which expand signaling capacity beyond membrane‐restricted receptor expression [96, 124]. IL6‐sIL6R dimers can bind full length membrane GP130 to stimulate intracellular signaling, whereas the trimeric complex containing IL6, sIL6R, and sGP130 sequesters IL6 and limits signaling, potentially inhibiting both trans‐ and cis‐signaling (Figure 2) [124, 125]. GP130 is widely expressed, but NSRs like IL6R are limited to some cell types. Thus, cells expressing GP130 but lacking IL6R may respond primarily via trans‐signaling [124]. Consequently, monoculture of cancer cells may underestimate the impact of BZA on GP130 signaling. Incorporating cocultures with stromal or immune cells is, therefore, crucial, as these cells contribute significantly to cytokine production in the tumor microenvironment [126].

### 5.3. Clinical Implications

BZA represents a mechanistically interesting modulator of GP130‐associated signaling in preclinical systems. Across models, it consistently affects IL6/IL11–linked pathways including STAT3, PI3K/AKT/mTOR, and MAPK, suggesting a reproducible pathway‐level signature. However, the clinical relevance of these findings remains to be established, as current evidence is derived primarily from experimental systems and has not been validated under pharmacologically guided in vivo conditions. Future studies integrating receptor expression profiling, tumor/tissue microenvironment complexity, and exposure–response relationships will be required to define its therapeutic potential.

## 6. Effect of BZA on ROS Production and Ferroptosis Involving ER, GP130, or Potential Other Targets

ERα, ERβ, and GPER regulate ROS through canonical and noncanonical mechanisms. ER signaling is generally associated with reduced ROS through antioxidant defenses and mitochondrial effects, although context‐dependent pro‐oxidant signaling has also been reported in proliferative settings [127–131]. ROS can in turn modulate NF‐κB activity and IL6 signaling, forming interconnected regulatory loops linking inflammation and redox homeostasis [132–134]. Dysregulation of these pathways contributes to diseases such as osteoporosis, osteoarthritis, and cancer [135–137].

Within this framework, BZA has been reported to modulate ROS levels, partly through its ER‐dependent effects and possibly through its GP130‐dependent effects. At low concentrations, it modulates cellular, mitochondrial, and lipid ROS across diverse models with varying ER expression (Table 3) [55, 138–155]. These effects were associated in some models with reduced STAT3 phosphorylation, suggesting possible involvement of GP130 or ER noncanonical signaling [146, 147, 149]. These findings position ROS modulation as a convergent downstream output of both ER and GP130/STAT3 signaling pathways rather than an isolated mechanistic effect. In arterial tissue from apolipoprotein E‐deficient mice, BZA increased nitric oxide synthase phosphorylation, an effect also observed with E2, consistent with ER‐linked redox regulation [156]. ER‐dependent signaling may involve SIRT1, as both E2 and BZA (2–6 μM) increased SIRT1 expression and its knockdown partially reversed BZA effect on ROS production and apoptosis in an endothelial cell line [148, 156]. However, in ER‐negative cells, BZA inhibited the formation of the E2 metabolite 4‐methoxyestrone (4‐MeOE1), further indicating the existence of an alternative mechanism independent of the receptor signaling [154, 157].

**TABLE 3:** Modulation of ROS by BZA across cell line models.

References	Model	Type of model	ER expression [138–145]	ROS induction	ROS method	BZA []	ROS modulation
*Cellular ROS*							
[146]	HCC827 et H1975 osimertinib resistant	Human lung adenocarcinoma cell lines	ERα: low ERβ: low	No induction	DCFH‐DA	2.5 and 5 µM	↑

[147]	H9c2	Rat myoblast cell line	ERα: no ERβ: yes	Isoproterenol	DCFH‐DA	5 µM	↓

[148]	HUVEC	Human endothelial cell line	ERα: no ERβ: yes	Angiotensin II	DCFH‐DA	2, 4, 6 µM	↓

[149]	H9c2	Rat myoblast cell line	ERα: no ERβ: yes	LPS	H2DCFDA	5 µM	↓

[150]	THP‐1	Human macrophage cell line	ERα: yes ERβ: yes	H37Ra infection	H2DCF‐DA	5 µM	NM

[151]	HUVEC	Human endothelial cell line	ERα: no ERβ: yes	TNF‐α	Dihydroethidium	4 µM	↓

[152]	MC3T3‐E1	Murine preosteoblast cell line	ERα: yes ERβ: yes	Homocysteine	Aminophenyl fluorescein	1 and 10 nM	↓

[153]	HUVEC	Human endothelial cell line	ERα: no ERβ: yes	AGEs‐BSA	CM‐H2DCFDA	10 nM	↓

[154]	MCF‐10A	Human breast cell line	ERα ERβ: low RNA levels	E2	CMH2DCFDA	1 μM	↓

[55]	HT22	Murine hippocampal cell line	ERα: yes ERβ: no	Erastin or RSL3	DCFH‐DA	1 μM	W/O induction = NM With induction = ↓
	HT22	Murine hippocampal cell line	ERα: yes ERβ: no	Erastin or RSL3	DAF‐FM‐DA	1 μM	W/O induction = NM With induction = ↓

*Mitochondrial ROS*							
[55]	HT22	Murine hippocampal cell line	ERα: yes ERβ: no	Erastin or RSL3	MitoSOX	1 µM	W/O induction = NM With induction = ↓

[150]	THP‐1	Human macrophage cell line	ERα: yes ERβ: yes	H37Ra infection	MitoSOX	5 µM	↑

*Lipid ROS*							
[55]	HT22	Murine hippocampal cell line	ERα: yes ERβ: no	Erastin or RSL3	C11 BODIPY 581/591	1 μM	W/O induction = NM With induction = ↓

[155]	HT‐1080	Human fibrosarcoma cell line	ERα: NR ERβ: yes	No induction or Erastin	C11 BODIPY 581/591	1 μM	W/O induction = NM With induction = ↓

*Note:* [] = BZA concentration, W/O = without, ↑ = increase, ↓ = decrease.

Abbreviations: ER = estrogen receptor, NM = no modification, NR = not reported, ROS = reactive oxygen species.

Because lipid peroxidation and ROS accumulation are hallmarks of ferroptosis, BZA’s redox effects may have functional consequences [158]. Within the integrated ER–inflammation–redox network described above, the modulation of ROS and lipid peroxidation provides a mechanistic bridge to ferroptosis regulation. In fact, BZA inhibits ferroptosis in several cell lines (80nM‐1.65 μM), with efficacy comparable to ferrostatin‐1 at similar concentrations [155, 158]. Among SERMs tested, only BZA and RAL showed this effect, and cotreatment with E2 did not alter BZA’s activity, supporting an ER‐independent activity [155]. However, upstream pathway involvement (e.g., GP130) has not been directly demonstrated and remains speculative [155].

**FIGURE 3:**
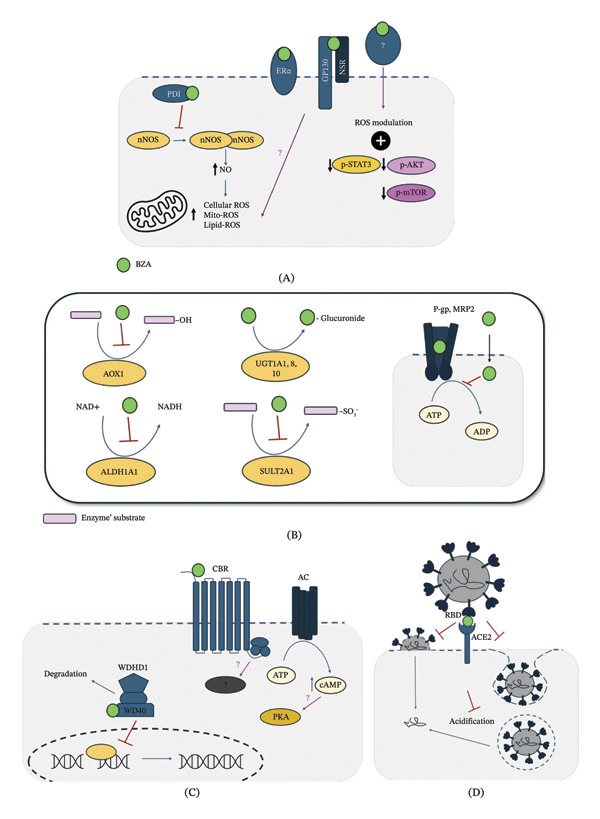
BZA interactions with putative targets. (A) BZA modulates reactive oxygen species (ROS) through multiple mechanisms. It binds PDI, inhibiting NOS dimerization and nitric oxide (NO) production, thereby reducing ROS levels. The contribution of ER‐dependent mechanisms remains unresolved, and in some models, BZA effects appear ER‐independent. Current evidence is also insufficient to determine whether GP130 signaling contributes to these effects. Although BZA alters signaling pathways such as p‐STAT3 and p‐AKT, the direct mediators of ROS modulation remain to be identified. (B) BZA inhibits the activity of biotransformation enzymes, including AOX1, ALDH1A1, and SULT2A1 and is metabolized via UGT‐mediated pathways. It is also transported by P‐glycoprotein (P‐gP) and MRP2 and inhibits their ATPase activity. (C) BZA binds the WD40 domain of WDHD1, promoting its degradation and impairing DNA repair. BZA also interacts with CBRs, leading to increased cAMP levels; however, its effects on other CBR‐associated signaling pathways remain unclear. (D) BZA has been reported to interact with the RBD–ACE2 complex in vitro and may inhibit SARS‐CoV‐2 infection. Red flat‐ended arrows indicate the inhibition by BZA. Fuchsia arrows indicate the unknown part of BZA mechanism. Abbreviations: BZA = bazedoxifene, AOX1 = aldehyde oxidase 1, ALDH1A1 = aldehyde dehydrogenase, UGT= UDP‐glucuronosyltransferase, SULT= sulfotransferase, P‐gp = P‐glycoprotein 1, MRP = multidrug resistance protein, WDHD1 = WD repeat and HMG‐box DNA‐binding protein 1, CBR= cannabinoid receptor, AC = adenylyl cyclase, PKA= protein kinase A, PDI= protein disulfide isomerase, nNOS= Neuronal nitric oxide synthase, NO= Nitric oxide, ROS= Reactive oxygen species, ER= estrogen receptor, GP130 = glycoprotein 130, p‐STAT3 = phosphorylated signal transducer and activator of transcription 3, p‐AKT = phosphorylated protein kinase B, p‐mTOR= phosphorylated mechanistic target of rapamycin, NSR= nonsignaling receptors, RBD = spike glycoprotein‐receptor binding domain, ACE2 = angiotensin‐converting enzyme 2.

Additional targets have been proposed to further explain BZA’s regulation of ROS and ferroptosis. BZA (1.25–20 μM) directly interacted and inhibited protein disulfide isomerase (PDI) activity in vitro, reducing nitric oxide synthase dimerization and attenuating erastin‐induced nitric oxide, mitochondrial ROS, and lipid ROS (Figure 3A) [55]. This interaction is supported by biophysical (Kd = 3.6 nM) and in silico approaches, and effects were observed in ER‐negative cells [55]. In vivo, neuroprotective effects of BAZ have been reported in association with reduced neuronal loss, consistent with PDI inhibition [55]. Although this target has been evaluated by a single group, they provided orthogonal validation supporting PDI as a candidate worthy of further investigation. While the reported binding affinity suggests that interaction with PDI could occur at a clinically relevant concentration, its contribution to BZA’s overall pharmacological effects remains uncertain.

Also, BZA inhibited 15‐lipoxygenase activity at high concentrations (50 μM), but this is likely not physiologically relevant based on *C*_max_ [152, 158]. In endothelial cells, BZA reduced CD40 expression and ROS‐associated inflammatory responses at 4 μM, but this observation is based on a single model and requires validation in additional systems [151].

Overall, current evidence suggests that BZA may influence ROS homeostasis and ferroptosis via a combination of ER‐dependent and ER‐independent mechanisms operating within an integrated ER‐GP130‐redox network. Among proposed mechanisms, PDI inhibition represents a plausible ER‐independent contributor, although its physiological relevance remains to be established. These findings support a potential role for BZA in modulating oxidative stress‐related processes but should be interpreted cautiously given the variability in experimental systems, concentrations used, and limited in vivo validation.

## 7. Effect of BZA on Cholesterol Synthesis Involving ER, GP130, or Potential Other Targets

Abnormal cholesterol synthesis contributes to cancer and cardiovascular diseases, making it a biologically and clinically pertinent process to target [159, 160]. Consistent with its established mechanism of action, BZA significantly reduces total and LDL cholesterol levels in women with or without osteoporosis, in line with the known role of ER signaling in lipid homeostasis [32, 161–163].

Beyond these ER‐mediated effects, preclinical studies have proposed additional targets involved in cholesterol biosynthesis, suggesting possible ER‐independent mechanisms. In skin fibroblasts from Smith–Lemli–Opitz patients (100 nM) and in mouse neuroblastoma cells deficient for 7‐dehydrocholesterol‐reductase (200 nM and 1 μM), BZA reduced 7‐dehydrocholesterol accumulation and increased desmosterol levels, an effect consistent with potential inhibition of 24‐dehydrocholesterol‐reductase (DHCR24). Comparable effects were observed with RAL and TAM in the same models [164]. However, these observations remain preclinical and mechanistically unresolved, as both STAT3 and ER are known to regulate DCHR24 transcription, and their contribution cannot be excluded without dedicated pathway‐specific assays [165].

Additional preclinical evidence supports ER‐independent activity. Indeed, in ERα/ERβ knockout mice, BZA (10 mg/kg/day) promoted remyelination and oligodendrocytes differentiation in the brain, suggesting the effects independent of canonical ER signaling [166]. Computational analyses further suggested that BZA may inhibit 3β‐hydroxysteroid‐Δ8,Δ7‐sterol isomerase (EBP). Following this prediction, cotreatment with BZA (500 nM) and a non‐EBP–inhibiting proremyelination compound enhanced oligodendrocyte differentiation compared to either treatment alone [166]. In vitro, TAM, BZA, and RAL increased the EBP substrate compared to control, indicating possible inhibition [164]. TAM has previously been reported to inhibit EBP [167]. However, these findings are currently hypothesis‐generating, as direct target engagement and specificity have not been experimentally demonstrated.

In summary, while BZA’s cholesterol‐lowering effects are clinically supported and primarily ER‐mediated, additional modulation of DHCR24 or EBP remains exploratory. Reported effects occur at concentrations (100 nM to low micromolar range) that partially meet the clinically achievable plasma levels but may also reflect tissue‐specific accumulation.

## 8. BZA Interactions With Metabolic and Transport Proteins

Drug biotransformation enhances elimination and can modify drug activity through three main processes [168]. Phase I oxidation, primarily mediated by cytochrome P450 enzymes (CYP), introduces polar groups that increase water solubility [168]. Phase II conjugation reactions such as glucuronidation and sulfonation catalyzed by UDP‐glucuronosyltransferase (UGT) and sulfotransferase (SULT) attach hydrophilic groups that further promote solubility and excretion [168]. Drug absorption also depends on lipid solubility and transport proteins such as pumps like P‐glycoprotein 1 (P‐gp), considered a Phase III process [169]. BZA may interact with all three phases, including metabolic enzymes and transport pumps, potentially influencing its absorption and clearance (Figure 3B).

### 8.1. Oxidation

BZA (25 μM) inhibited the oxidation of aldehyde oxidase 1 (AOX1)’s substrate in the presence of recombinant AXO1 and with human cytosol from liver or kidney, an effect also seen with other SERMs [53]. In liver cytosol, BZA showed strong inhibition with an IC50 of 0.19 ± 0.02 μM. *In silico* analyses suggested that BZA acts as a potential competitive inhibitor by interacting with key amino acid residues within the AOX1 active site [53]. Preclinical data in rats show that, following the oral administration of radiolabeled BZA, the liver radioactivity level was up to approximately 55‐fold higher than plasma levels, suggesting substantial hepatic uptake [39]. Given that AOX1 inhibition occurs at a concentration approaching clinically achievable levels (10‐fold higher than *C*_max_), particularly in the liver, this interaction may be considered potentially pharmacologically relevant, although in vivo confirmation is still pending.

*In silico* screening further proposed that BZA also interacts with aldehyde dehydrogenase (ALDH1A1) by binding key amino acid residues within its active site. In vitro enzymatic assay revealed that BZA selectively inhibited ALDH1A1 activity (IC50 = 4.41 ± 0.50 μM), while much higher concentrations are required to affect other ALDH isoforms [49]. Similar interactions with ALDH1A1 have been reported for other SERMs such as RAL and TAM [49, 170]. Although seemingly selective, the concentration of BZA required to meaningfully affect this enzyme activity is substantially higher than the clinically observed concentration (∼300‐fold higher), making the translation of this effect in humans unlikely, even when considering potential hepatic accumulation.

Unlike RAL metabolites, which inhibit CYP3A4, BZA (50 μM) did not behave as a substrate for major CYP isoenzymes in liver microsomes [171]. However in a breast model, RAL did not alter expression and activity of CYP1A1 and 1B1, indicating that a SERM may interact with specific CYP enzymes, the expression of which can vary between tissues [154]. In silico predictions suggested that BZA is a CYP substrate rather than an inhibitor, consistent with findings in human intestinal microsomes [172]. Indeed, unchanged BZA (0.1 μM) gradually disappeared over time when exposed to CYP450‐activated intestinal microsomes indicating potential metabolism by specifically intestinal enzymes [173]. Overall, these findings remain inconclusive and preclinical. Furthermore, the low urinary levels of BZA–N‐oxide relative to glucuronide metabolites in men receiving 20‐mg BZA suggest that oxidation is a minor pathway in humans [174].

### 8.2. Glucuronidation and Sulfonation

Glucuronidation is the clinically supported primary pathway of BZA metabolism. Two metabolites were predominant in the plasma of postmenopausal women following BZA uptake: BZA‐5‐glucuronide and BZA‐4′‐glucuronide [175]. BZA and RAL share a similar metabolic profile, as both are primarily metabolized by UGT1A1, UGT1A8, and UGT1A10 [173, 176, 177]. TAM metabolites are also substrates of UGT1A8 and UGT1A10 [178].

BZA (1 μM) additionally inhibits sulfonation, by acting as a noncompetitive inhibitor of SULT2A1. More specifically, BZA inhibited SULT2A1‐mediated lithocholic acid sulfonation both in human liver cytosol (IC50 = 0.21 ± 0.03 μM) and with human recombinant SULT2A1 [179]. At similar concentrations, BZA also inhibited sulfonation of other SULT2A1 substrates. As these in vitro concentrations were close to clinically achievable exposure in humans, this interaction may be considered potentially clinically relevant. Although, its in vivo consequences remain to be established. RAL and TAM were also identified as SULT2A1 inhibitors, with BZA and RAL showing greater potency than TAM [179, 180].

### 8.3. Transport of BZA

Due to its lipophilic nature, BZA can passively cross cell membranes [172, 173, 181, 182]. In addition, preclinical studies suggest that BZA may also be transported by P‐gp [176, 181]. Targeted P‐gp inhibition partially reduced BZA transport, indicating a contribution of both passive diffusion and active transport. Without P‐gp inhibition, increasing BZA concentrations (0.7–70 μM) decreased the exchange ratio, suggesting the saturation of P‐gp–mediated transport [176]. Furthermore, BZA (0.38–48 μM) inhibited the ATPase function of activated P‐gp (IC50 = 5 μM) and other transporters: multidrug resistance protein (MRP1 and MRP2; IC50 = 4.5 and 6 μM) and ATP‐binding cassette superfamily G member 2 (BCRP; IC50 = 9 μM). Inhibition was further confirmed for MRP2, as BZA (10 μM) decreased the transport of a well‐known substrate of the protein [181]. TAM and RAL also interact with P‐gp [183, 184]. Although BZA effects on active transport are dose‐dependent, concentration values of in vitro assays are higher than the maximum serum concentration of the standard dose. An in silico analysis also hypothesizes that BZA is a substrate for the renal organic cation transporter 2 (OCT2) [172]. However, this remains hypothesis‐generating and requires experimental validation.

### 8.4. Clinical Implications

Clinically and preclinical relevant targets of BZA related to metabolic enzyme interaction, hepatic enzyme modulation, and/or competition with endogenous substrates could influence BZA’s metabolic stability, systemic exposure, and elimination.

More specifically, the inhibition of AOX1 may alter the metabolism of endogenous substrates such as vitamin A and potentially increase the risk of drug–drug interactions, since AOX1 can oxidize or reduce a variety of drugs (e.g., allopurinol and clonazepam) [185–187]. Conversely, AOX1 inhibition could also be beneficial in certain contexts, as its activity has been linked to ROS production, and some of its metabolites have been associated with potential hepatotoxicity [185, 187].

Regarding interactions with SULT2A1, a key enzyme involved in steroid homeostasis and bile acid detoxification, potential adverse hepatic effects should be carefully evaluated in future clinical repurposing studies, particularly at higher dosage [179]. Given the low micromolar potency observed in vitro, this interaction may be of potential pharmacological relevance, although in vivo validation is required.

Finally, interactions involving active transporters have been validated preclinically but appear physiologically unlikely at the authorized dosing. However, if higher concentrations of BZA are explored in clinical settings, these interactions should be further investigated, as they may influence the absorption and clearance of BZA and coadministered drugs, particularly in tissues or disease where these transporters contribute to multidrug resistance [188]. Collectively, these findings underscore the importance of considering metabolic enzyme and transporter profiles when optimizing BZA dosing, anticipating pharmacokinetic variability, and evaluating combination therapies.

## 9. BZA Interaction With WD Repeat and HMG‐Box DNA‐Binding Protein 1 (WDHD1)

Cell‐cycle control and DNA repair are crucial for maintaining cellular homeostasis. Alteration of these processes contributes to cancer progression, treatment resistance, and other disorders like diabetic kidney disease [189, 190].

BZA may interfere with DNA repair, replication, and cell‐cycle progression through WDHD1 (Figure 3C). BZA reduced WDHD1 expression in the low micromolar range (1–10 μM; IC50 = 0.32 μM), while WDHD1 overexpression restored cell viability, supporting a functional BZA–WDHD1 interaction [11, 191]. This effect appeared ERα‐independent and was observed across multiple cancer types including breast, ovarian, and colorectal cancers [191]. Cellular thermal shift assay and docking analyses further suggested direct binding, likely involving the WD40 domain of WDHD1 [11, 191]. Consistently, BZA (1 and 4 μM) impaired WDHD1‐mediated DNA repair by reducing homologous recombination efficiency and CtBP‐interacting protein recruitment following DNA damage [191, 192]. It also enhanced cisplatin sensitivity in vitro (1 μM) and reduced tumor growth in combination with cisplatin and inhibited WDHD1 levels in vivo (2 mg/kg/3 days) [191]. Both BZA treatment and WDHD1 depletion decreased the S‐phase cell population, supporting a tumor‐suppressive effect of BZA mediated at least in part through WDHD1 inhibition [191].

Altogether, preclinical data suggest that BZA may modulate DNA repair and cell‐cycle progression through WDHD1, notably by promoting its degradation and impairing homologous recombination. This mechanism is of particular interest in oncology, as the activation of DNA repair pathways is a recognized driver of resistance to DNA‐damaging therapies [189]. However, the translational relevance of this interaction requires cautious interpretation, as the reported IC50 remains above the clinically achievable systemic exposure with current dosing (approximately 20‐fold higher).

In addition, *WDHD1* is a target gene of STAT3 and AKT, highlighting the need to further explore BZA’s crosstalk with GP130‐related signaling pathways [193, 194]. Further studies will be required to determine whether WDHD1 represents a direct pharmacological target of BZA in vivo or reflects downstream pathway crosstalk. The specificity of this interaction relative to other SERMs also remains to be clarified.

## 10. BZA Interactions With Cannabinoid Receptors (CBRs)

Off‐target effects of TAM in breast cancer treatment may involve CBR signaling, prompting the investigation of other SERMs like BZA [195]. CB1R is primarily expressed in the nervous system and adipose tissue, whereas CB2R predominates in immune and reproductive tissues [196]. Both are G protein–coupled receptors that regulate downstream effectors such as adenylyl cyclase and are currently investigated as potential targets in various diseases, including cancers and neurodegenerative diseases [197, 198].

BZA binds CB1R with moderate affinity (Ki = 836 nM) and CB2R with higher affinity (Ki = 253 nM) and displays inverse agonist properties in vitro [199]. With CB1R, BZA reduces G‐protein activity, antagonizes agonist‐induced signaling, and increases cyclic adenosine monophosphate (cAMP) levels at micromolar concentrations [199]. Through its interaction with CB2R, BZA inhibits ligand binding and suppresses G‐protein activity, with functional inhibition observed in the nanomolar range (IC50 = 71.6 nM), also accompanied by an accumulation of cAMP and the capacity to interfere with agonist‐induced cAMP inhibition [199, 200].

Collectively, these findings indicate that BZA acts as an inverse agonist of both CB1R and CB2R in vitro, with higher affinity for CB2R (Figure 3C). Notably, reported activity with CB2R occurs within the nanomolar to low submicromolar range (fivefold higher than the *C*_max_), making this protein a plausible physiological target of BZA. BZA–CB2R interaction is also supported by relatively strong evidence, with multiple lines of investigation reported across two models. Other SERMs like RAL and TAM metabolites also target CBRs, although TAM lacks CB2R selectivity [195, 199]. Overall, CB2R represents a mechanistically supported but still underexplored target of BZA that warrants further investigation in physiologically relevant models.

## 11*. In Silico*–Predicted Interactions of BZA With Human Proteins

### 11.1. Human Ether‐a‐go‐go Potassium Channel (hERG)

hERG inhibition is associated with arrhythmias and an increased risk of cardiac death and is therefore routinely assessed during drug development [201, 202]. For BZA, in silico predictions suggest a low likelihood of hERG liability. Estimated inhibitory concentrations are in the low micromolar range (0.52 μM), and the predicted QPlogHERG value falls below the accepted threshold of −5, indicating low risk of cardiac toxicity [45, 203]. Additionally, a third computational analysis did not identify BZA as an hERG inhibitor [172]. However, these assessments are based exclusively on computational models, and no dedicated in vitro nor in vivo studies have directly evaluated hERG inhibition by BZA to our knowledge. Notably, other SERMs, including TAM and RAL, have demonstrated hERG inhibition in vitro, suggesting that class‐related effects cannot be excluded [204, 205]. Overall, while current data point toward a low predicted cardiac liability, experimental validation is required to confirm the cardiac safety profile of BZA.

### 11.2. Beta‐Secretase 1 (BACE‐1)

Molecular docking studies suggest that BZA and RAL may bind BACE‐1, a key enzyme involved in amyloid plaque formation, with predicted affinities comparable to another known inhibitor (EJ7) [38]. However, BZA (0.0001–10 μM) had no functional effect in HEK293 expressing BACE‐1 and its substrate [84]. These findings do not support functional inhibition under the tested conditions, and the relevance of this interaction remains to be established.

### 11.3. Immune‐Related Proteins

In addition to inhibiting IL6‐related signaling pathways, BZA has been predicted in silico to interact with homodimer of programmed death‐ligand 1 (PD‐L1), N‐terminal domain of PD‐L1, N‐terminal domain of cytotoxic t‐lymphocyte–associated protein 4 (CTLA‐4), and N‐terminal domain of programmed cell death protein 1 (PD‐1) [50]. These proteins are targets of immunotherapy, and their inhibition can allow the immune system to recognize cancerous cells [206]. The estimated BZA concentrations needed for the predicted binding affinity (Ki) ranged from 14.3 μM to 33.74 μM, which exceed clinically reached plasma concentration [50]. While docking scores were more favorable for BZA than for RAL or TAM, these predictions have not been validated experimentally [50]. BZA–CD80 binding was also investigated in silico, but docking score and Ki were very high, indicating a low probability for this interaction to occur in the biological context [50]. Taken together, current data do not support functional engagement of these immune targets under physiological conditions. In vitro assays are required to determine whether BZA truly interacts with these proteins.

### 11.4. Casein Kinase 2 (CK2)

CK2 is a multifunctional kinase that regulates multiple signaling pathways, including the DNA damage response, PI3K/AKT, JAK2/STAT3, and androgen receptor pathways [207]. Molecular docking studies suggest that BZA may bind CK2 with predicted affinity comparable to or slightly stronger than a known inhibitor with similar hydrophobic interactions at key residues [52]. Nonetheless, molecular dynamics stimulation indicated that BZA and CK2 formed an unstable complex, suggesting limited structural robustness of the interaction [52]. Notably, similar in silico kinase interactions have been reported for other SERMs, and TAM has previously been characterized as a CK2 inhibitor in vitro [208]. Overall, these findings suggest a possible class‐related, but structurally weak, interaction that requires experimental validation to establish functional relevance.

### 11.5. Clinical Implications

Overall current in silico analyses of BZA suggest a limited likelihood of clinically relevant off‐target interactions at the approved dose. Safety pharmacology predictions, including hERG channel and immune checkpoint proteins (PD‐1, PD‐L1, CTLA‐4, CD80), indicate either low predicted affinity or micromolar‐range interactions exceeding *C*_max_ of authorized dose. While no experimental studies have confirmed these effects for BZA, the absence of reported cardiac adverse events in clinical use is consistent with a low predicted hERG liability [5].

Enzyme and kinase targets, including BACE‐1 and CK2, are supported by docking‐based predictions but not by consistent functional or structural validation. BACE‐1 activity was not affected in cellular assays, while CK2 interactions appear unstable in molecular dynamics simulations, suggesting limited functional relevance.

Overall, these computationally derived interactions should be considered exploratory rather than established pharmacological effects of BZA with pending targeted experimental validation.

## 12. Nonhuman Proposed Targets of BZA

### 12.1. SARS‐CoV‐2

In 2020, the COVID‐19 pandemic triggered an unprecedented surge in research on SARS‐CoV‐2 infection. IL6 was identified as a potential therapeutic target due to its elevated levels in infected patients [209]. Because BZA inhibits IL6‐related signaling pathways in various experiments, it was subsequently investigated as a candidate for drug repurposing, leading to the identification of additional potential molecular targets. In vitro studies demonstrated that BZA inhibits SARS‐CoV‐2 infection across multiple models, with IC50 values ranging from approximately 2 μM to 13 μM [210–212].

These antiviral effects are primarily attributed to interference with viral entry [213] (Figure 3D). Computational modeling predicted that BZA binds to key residues at the interface between the spike glycoprotein‐receptor binding domain (RBD) and the host receptor angiotensin‐converting enzyme 2 (ACE2) [54]. Experimentally, preincubation of ACE2 with BZA reduced RBD binding, with an IC50 of 1.237 μM[54]. Consistently, membrane fusion assays showed that BZA pretreatment (10 μM) inhibited fusion between ACE2‐expressing and spike‐expressing cells [211]. BZA was also reported to alter viral entry‐related processes by increasing intracellular cholesterol accumulation and promoting endosomal acidification [211]. In vivo, BZA reduced viral load and decreased IL6 expression [211]. In comparison, RAL can interact with the spike protein but appears not to interfere with the spike‐ACE2 binding [214]. An additional in silico study suggested possible BZA binding to the viral main protease (M^pro^), although this interaction has not been experimentally confirmed [41].

In summary, BZA inhibits SARS‐CoV‐2 infection in vitro and likely interferes with viral entry, primarily through the modulation of the RBD‐ACE2 interaction. However, the reported activities occur at micromolar concentrations, and their relevance under clinically achievable exposure conditions remains to be established. Although BZA has also been tested against other viral infections, the specific molecular targets underlying its broader antiviral effects have not been fully elucidated [215–219].

### 12.2. *Leishmania donovani*–Related Proteins

*L. donovani* is a pathogenic parasite responsible for leishmaniasis. *In silico* analyses identified BZA as a potential inhibitor of citrate synthase enzyme (*L. donovani* citrate synthase enzyme [LdCS]), a key enzyme of the TCA cycle [172]. BZA showed a more favorable predicted binding energy for LdCS than for the human enzyme, suggesting potential selectivity. Predicted interactions with key binding site residues and an inhibitor constant in the nanomolar‐range further support LdCS as a candidate target. Functionally, BZA (3 μM) reduced extracellular parasite viability by 90% and 2.11 ± 0.38 μM BZA inhibited by 50% the number of intracellular parasites in a murine macrophage. However, limited selectivity was observed, as the drug also reduced by half the viability of the macrophage cell line at 11.8 ± 0.56 μM, precluding further analysis [172].

Other in silico data suggest that BZA may also target *L. donovani* glycyl‐tRNA synthetase (LdGlyRS), which is an enzyme essential for protein synthesis, and form a stable complex through interactions with key residues [51]. Overall, while these findings indicate potential antiparasitic activity, the observed effects occur at micromolar concentrations and are accompanied by host cell toxicity, leaving the therapeutic significance to be clarified.

### 12.3. *Staphylococcus aureus* Glycolytic Enzymes

In the search for selective antibacterial compounds, in silico screening identified BZA as a potential interactor of glycolytic enzymes of *S. aureus*. BZA had a ChemPLP score of approximately 90 for a known allosteric site of phosphofructokinase and for a newly proposed allosteric site of pyruvate kinase. However, BZA was not included among the top candidates selected for functional validation, and no experimental data are available to confirm these interactions [220]. As such, the relevance of these predicted targets remains undetermined.

### 12.4. Clinical Implications

Preclinical data suggest that BZA may exert antiviral and antiparasitic effects, although its translational potential remains uncertain. Interactions with lung‐specific proteins warrant further examination, particularly in light of in vivo findings suggesting that BZA can accumulate in pulmonary tissue [39]. However, predicted human tissue distribution and antiviral activity observed in a monkey cell line suggested that BZA is unlikely to be effective against COVID‐19 when administered via its conventional oral route [221]. Importantly, these conclusions drawn from a single BZA–SARS‐CoV‐2 study do not preclude its potential utility under alternative delivery strategies or formulations designed to improve tissue targeting and bioavailability. BZA appears to interact with both ACE2 and LdCS at low micromolar concentrations, which are approximately 100‐fold higher than *C*_max_, further indicating that *per os* administration of the currently approved dose would make BZA interactions with these targets improbable.

In addition, achieving meaningful viral or parasitic inhibition may require concentrations that are also toxic to host cells. Moreover, because BZA interacts with several human proteins at submicromolar to low micromolar levels in vitro, efforts to target parasites may cause off‐target effects.

For bacterial targets, including glycolytic enzymes of *S. aureus*, current evidence is restricted to in silico predictions without functional validation, precluding the assessment of biological pertinence. However, this study opens new avenues for exploring the effects of BZA on allosteric sites rather than limiting the analysis to enzyme ligand‐binding sites.

Taken together, these findings indicate that the nonhuman targets identified for BZA are supported by limited or context‐dependent evidence and generally occur at exposure levels exceeding those achieved with standard dosing. While alternative delivery strategies or optimized formulations could theoretically enhance tissue exposure, their impact on target engagement and safety remains to be determined.

## 13. Discussion/Conclusion

### 13.1. Critical Evaluation

This review identified more than 30 known or literature‐proposed BZA interactors. However, only five (ERα, UGT, CB2R, GP130, and PDI) reached a high level of evidentiary strength according to our critical framework (Table 1 and Supporting Table 1). Among these, ERα and UGT are supported by clinical evidence, whereas the remaining three proteins are underpinned by robust preclinical data and/or interstudy replication/orthogonal validation. In addition, seven interactors (ERα, GP130, AOX1, ALDH1A1, WDHD1, RBD/ACE2, and PDI) are supported by convergent lines of evidence, integrating in silico predictions, in vitro direct or indirect interaction assays, and functional validation in vitro or in vivo. We also identified targets (ERβ, AOX1, and SULT2A1) for which the current level of evidence is moderate, although reported effects may happen at physiological concentration. Thus, despite a growing body of literature, robust mechanistic validation remains limited for most proposed targets.

Even among well‐supported interactions, important gaps persist. While BZA’s effects on canonical ERα are well characterized, its engagement with membrane‐bound ERα, ERβ and ERα/ERβ heterodimers remains insufficiently characterized [63, 77, 83, 87]. More broadly, the molecular determinants of BZA tissue selectivity and cofactor specificity are still poorly understood [42]. Addressing these questions is not merely of mechanistic interest: A deeper understanding could enable refined patient stratification, optimize SERM selection, and guide both drug repurposing efforts and the rational design of next‐generation SERMs and SERDs with improved efficacy and signaling selectivity.

Similarly, multiple studies have proposed that BZA, through binding to the first domain of GP130, modulates the assembly of IL6‐ or IL11‐associated receptor hexamers, thereby inhibiting downstream signaling pathways including STAT3, PI3K/AKT/mTOR, and MAPK [10, 114]. However, these effects are predominantly observed at supraphysiological concentrations, raising concerns regarding their translational relevance at the approved 20‐mg dose. While higher dosing does not directly establish target engagement, data in humans indicate that it might be safe for administration. Indeed, 40 mg/day maintains a safety profile similar to 20 mg over 3 years, and 80 mg daily is well tolerated over 30 days, suggesting that dose escalation may be feasible within defined timeframe [21, 37]. Notably, a 37% increase in apparent volume of distribution with 40 mg daily indicates the potential for greater tissue accumulation [37]. These observations suggest that interactions happening at higher concentrations in in vitro or in vivo models, such as those involving GP130, cannot be entirely excluded and deemed as physiologically unachievable with safe doses of BZA. Under such conditions, targeting GP130‐mediated signaling could merit further investigation, alongside other targets reported to be modulated within similar concentration ranges. Although higher doses may increase systemic and tissue exposure, the relationship between dose, tissue concentration, and target engagement remains to be formally established. In parallel, any strategy involving higher exposure or molecular optimization will require careful evaluation of safety liabilities, including hepatic metabolism, drug–drug interactions, and cardiotoxicity.

For many remaining proposed targets, the level of evidence remains low, with no confirmation in vitro or in vivo. Several interactions, including M^pro^, CK2, hERG, LdGlyRS, phosphofructokinase, pyruvate kinase, and immune‐related proteins (PD‐L1, CTLA‐4, and PD‐1), are suggested in the literature exclusively based on in silico predictions (Table 1) [41, 45, 50–52, 172, 203, 220]. While valuable for hypothesis generation, such approaches do not account for protein dynamics, pharmacokinetics, cellular compartmentalization, or the functional consequences of binding. Accordingly, the pharmacological relevance of these interactions remains uncertain, and whether they occur at clinically achievable concentrations of BZA has yet to be established.

### 13.2. In Vitro Considerations

Interpretation of in vitro data requires caution across both low and high micromolar concentrations. At lower concentrations, several proposed BZA interactions exhibit dose‐dependent effects in the nanomolar to low micromolar range warranting further validation to confirm their reproducibility and biological relevance. Thus, a careful discussion of in vitro/in vivo transcriptomic or phenotypic findings, particularly in ER‐negative models, will be necessary in future studies.

At higher concentrations, additional limitations emerge. BZA behaves as a colloidal aggregator at micromolar concentrations, with a critical aggregation threshold of approximately 26 μM [222]. Colloidal aggregation is a well‐recognized experimental artifact in drug screening, as aggregates can nonspecifically adsorb proteins, leading to artificial interactions and false‐positive results [222, 223]. Accordingly, findings obtained at or above this threshold should be interpreted carefully. To enhance transparency, we reported BZA concentrations across studies and explicitly integrated concentration ranges into our critical framework, thereby strengthening the overall assessment of evidence supporting each proposed target.

### 13.3. BZA Target Profile, in Comparison With Other SERMs

Although many BZA targets overlap with those of TAM and RAL, functional distinctions may confer therapeutic advantages (Table 4). Notably, TAM‐resistant breast cancer cell lines remain responsive to BZA despite harboring mutations in the AF2 domain, suggesting that BZA may serve as an effective alternative to TAM [70]. Nevertheless, other AF2 mutations may alter BZA function, underscoring the need for a personalized approach to assess patient mutation profiles and guide SERM selection.

**TABLE 4:** BZA protein interactions with high and moderate strength of evidence compared to two other SERMs: RAL and TAM.



*Note:* Yellow boxes indicate that there is evidence of SERM interactions with the protein. Light yellow indicates that the SERM has been tested, but the interaction was reported as not probable. Undocumented interactions are represented by white boxes.

Abbreviations: BZA = bazedoxifene, RAL = raloxifene, SERM = selective estrogen receptor modulator, TAM = tamoxifen.

Beyond ER signaling, differences in noncanonical targets may further distinguish these agents. Unlike TAM, for which there is no report of GP130 interaction, both RAL and BZA can interact with this signaling receptor in vitro. Importantly, the concentrations required to achieve comparable modulation of GP130‐mediated pathways are lower for BZA (20 μM) than for RAL (50 μM) [10]. While these findings remain within the micromolar range and therefore require cautious interpretation, they suggest that BZA may achieve similar biological effects at lower exposure levels under certain conditions.

### 13.4. Conclusions and Future Directions

This review provides a comprehensive and critical synthesis of both established and emerging BZA targets, integrating mechanistic insights that extend beyond prior disease‐centric analyses. By broadening the framework to include noncanonical ER signaling, heterodimerization, cofactor specificity, and GP130‐mediated cytokines network beyond IL6, we propose a more unified view of BZA pharmacology. We also present the first consolidated discussion linking BZA to ROS production and ferroptosis, thereby expanding its potential relevance to diseases characterized by oxidative stress.

Collectively, these findings refine the current understanding of BZA’s molecular landscape while emphasizing the need for rigorous binding and functional validation of emerging targets. Beyond delineating its therapeutic scope, this work may inform the rational development of next‐generation SERMs optimized not only for hormone‐responsive cancers and osteoporosis but also for inflammatory, redox‐driven, and potentially viral diseases.

**Abbreviations**
15‐LOX: 15‐Lipoxygenase
AF1: Activation function‐1
AF2: Activation function‐2
ACE2: Angiotensin‐converting enzyme 2
AKT: Protein kinase B
ALDH1A1: Aldehyde dehydrogenase
AOX1: Aldehyde Oxidase 1
BCRP: ATP‐binding cassette superfamily G member 2
BZA: Bazedoxifene
BACE‐1: Beta‐secretase 1
CBRs: Cannabinoid receptors
cAMP: Cyclic adenosine monophosphate
CE: Conjugated estrogen
CEE: Conjugated equine estrogen
CK2: Casein kinase 2
Cmax: Maximum plasma concentration
CTLA‐4: Cytotoxic T‐lymphocyte–associated protein 4
CYP: Cytochrome P450 enzymes
DHCR24: 24‐Dehydrocholesterol‐reductase
E2: Estradiol
EBP: 3β‐hydroxysteroid‐Δ8,Δ7‐sterol isomerase
EC10: Estrogen compounds
ER: Estrogen receptor
ERK2: Extracellular signal‐regulated kinase 2
GP130: Glycoprotein 130
GPER: G protein–coupled estrogen receptor
hERG: Human ether‐a‐go‐go potassium channel
IC50: Median inhibitory concentration
IL11: Interleukin 11
IL11R: Interleukin 11 receptor
IL6: Interleukin 6
IL6R: Interleukin 6 receptor
LAS: Lasofoxifene
LdCS: *Leishmania donovani* citrate synthase enzyme
LdGlyRS: *Leishmania donovani* glycyl‐tRNA synthetase
LIF: Leukemia inhibitory factor
LIFR: LIF receptor
MAPK: Mitogen‐activated protein kinase
M^pro^
: SARS‐CoV‐2 main protease
MRP: Multidrug resistance protein
NSR: Nonsignaling receptors
OCT2: Organic cation transporter 2
OSM: Oncostatin M
OSMR: OSM receptor
OVX: Ovariectomized
P‐gp: P‐glycoprotein 1
PI3K: Phosphoinositide 3‐kinase
PD‐1: Programmed cell death protein 1
PDI: Protein disulfide isomerase
PD‐L1: Programmed death‐ligand 1
RAL: Raloxifene
RBD: Spike glycoprotein‐receptor binding domain
ROS: Reactive oxygen species
SERD: Selective estrogen receptor degrader
SERM: Selective estrogen receptor modulator
STAT3: Signal transducer and activator of transcription 3
SULT: Sulfotransferase
TAM: Tamoxifen
UGT: UDP‐glucuronosyltransferase
WDHD1: WD repeat and HMG‐box DNA‐binding protein 1

## Author Contributions

Juliette Bherer: conceptualization, investigation, visualization, writing–original draft, and writing–review and editing.

Alisson Clemenceau: conceptualization, supervision, writing–original draft, and writing–review and editing.

Caroline Diorio: supervision and writing–review and editing.

Francine Durocher: supervision and writing–review and editing.

## Funding

Juliette Bherer holds a Doctoral Research Award: Canada Graduate Scholarships from the Canadian Institutes of Health Research (203323) at the time of the revision and held a doctoral research award from the Cancer Research Society (1332504) and a doctoral training scholarship from the Fonds de Recherche du Québec‐Santé (351275) during the investigation and writing of the original draft. Alisson Clemenceau holds a Leslie H. Warner postdoctoral fellowship for cancer research from the Yale Cancer Center.

## Conflicts of Interest

The authors declare no conflicts of interest.

## Supporting Information

Additional supporting information can be found online in the Supporting Information section.

## Supporting information


**Supporting Information** File titled Supporting _Table_1_2_and_3_final (word): Supporting Table 1. Detailed criteria and classification framework used to assess the strength of evidence and the conclusions based on both the strength of evidence and the information for each proposed BZA interactors. Supporting Table 2. Docking score values of BZA targets and/or potential targets found in the literature. Supporting Table 3. Gene chip and RNAseq detailed data of cell lines and tissues treated with BZA alone or in combination with estrogen compounds (E2, CE, EC10, or CEE).

## Data Availability

Data sharing is not applicable to this article as no datasets were generated or analyzed during the current study.
