# Supplementary material for: Bazedoxifene and Beyond: Identifying This SERM’s Targets and Deciphering Its Molecular Mechanisms
Source: Adv Pharmacol Pharm Sci. 2026 Aug 4;2026:3028716. doi: 10.1155/adpp/3028716 (PMC13435104; doi:10.1155/adpp/3028716)
Supplement: Supplementary file 1 [file ADPP-2026-3028716-s001.docx]

**Supporting Table 1. Detailed criteria and classification framework used to assess the strength of evidence and the conclusions based on both the strength of evidence and the information for each proposed BZA interactors.**

| **Protein** | **Experimentally determined binding?** | **Functional impact?** | **Functional effect at relevant concentrations?** | ***In vivo* testing** | **Number of supporting studies** | ***In silico* data?** | **Clinical relevance supported** | **Results** | **Conclusions based on strength of evidence and information relative to Cmax (see description in blue)** | |
| --- | --- | --- | --- | --- | --- | --- | --- | --- | --- | --- |
| Scores and description of evaluated domains | 3: Multiple direct binding assays (e.g., radioligand binding) or one binding assay supported by an additional independent method (orthogonal validation or cross-study validation) | 3: Clear dose-dependent functional modulation (e.g., enzymatic assay, reporter gene transcription, etc.) | 2: nM ranges | 1: Yes | 3: More than two studies supporting the findings | 1: *In silico evidence* for either binding or functional prediction only | 1: Confirmed in humans | high: 10 to 14 | **IC50 (or Kd or Ki)/ Cmax:**  <10X difference → plausible clinical relevance  10–100X → uncertain clinical relevance/ tissue-dependent  >100X → unlikely | |
|  | 2: Single direct binding assay (e.g. radioligand, DARTS, CETSA, etc.) | 2: Functional effect at set doses with phenotype linked to mechanism (e.g., KO, siRNA, shRNA, co-treatment) | 1: 1-10µM | 0: No | 2: Two studies supporting the findings | 0: No *in silico* data available | 0: Not confirmed in humans | moderate: 6 to 9 |  | |
|  | 1: Indirect evidence of binding (assay with only recombinant protein, with a known inhibitor of this protein tested with more than one concentration) | 1: Phenotypic effect observed without clear mechanistic link | 0: >10µM or not applicable |  | 1: Single study reported |  |  | weak: 0 to 5 |  | |
|  | 0: No evidence of binding | 0: No functional evidence reported |  |  | 0: conflicting or divergent conclusions between studies |  |  |  |  | |
|  |  | -1: Tested but no effect observed |  |  |  |  |  |  |  | |
| ERα | 3 | 3 | 2 | 1 | 3 | 1 | 1 | 14 | | **Clinical relevance supported;** Plausible clinical relevance |
| UGT (1A1,1A8 and 1A10) | 1 | 3 | 2 | 1 | 3 | 0 | 1 | 11 | | **Clinical relevance supported** |
| CB2R | 3 | 3 | 2 | 0 | 2 | 0 | 0 | 10 | | Preclinical experimental supported; Plausible clinical relevance |
| GP130 | 2 | 2 | 1 | 1 | 3 | 1 | 0 | 10 | | Preclinical experimental supported |
| PDI | 3 | 3 | 1 | 1 | 1 | 1 | 0 | 10 | | Preclinical experimental supported; Plausible clinical relevance |
| WDHD1 | 2 | 2 | 1 | 1 | 2 | 1 | 0 | 9 | | Preclinical experimental supported; Uncertain clinical relevance/ tissue-dependent |
| RBD/ACE2 | 2 | 1 | 1 | 1 | 3 | 1 | 0 | 9 | | Preclinical experimental supported; Uncertain clinical relevance/ tissue-dependent |
| AOX1 | 1 | 3 | 2 | 0 | 1 | 1 | 0 | 8 | | Preclinical experimental supported; Plausible/uncertain clinical relevance |
| P-gp | 1 | 3 | 1 | 0 | 2 | 0 | 0 | 7 | | Preclinical experimental supported; unlikely |
| SULT2A1 | 1 | 3 | 2 | 0 | 1 | 0 | 0 | 7 | | Preclinical experimental supported; Plausible/uncertain clinical relevance |
| CB1R | 2 | 3 | 1 | 0 | 1 | 0 | 0 | 7 | | Preclinical experimental supported; Uncertain clinical relevance/ tissue-dependent |
| ALDH1A1 | 1 | 3 | 1 | 0 | 1 | 1 | 0 | 7 | | Preclinical experimental supported; unlikely |
| ERβ | 3 | 0 | 0 | 0 | 3 | 0 | 0 | 6 | | Preclinical experimental supported; Plausible clinical relevance |
| MRP2 | 1 | 3 | 1 | 0 | 1 | 0 | 0 | 6 | | Preclinical experimental supported; unlikely |
| MRP1 or BCRP | 1 | 3 | 1 | 0 | 1 | 0 | 0 | 6 | | Preclinical experimental supported; unlikely |
| EBP | 0 | 2 | 2 | 0 | 2 | 0 | 0 | 6 | | Hypothesis-generating only |
| DHCR24 | 0 | 2 | 2 | 0 | 1 | 0 | 0 | 5 | | Hypothesis-generating only |
| 15-LOX | 1 | 2 | 0 | 0 | 1 | 0 | 0 | 4 | | Hypothesis-generating only |
| CD40 | 0 | 2 | 1 | 0 | 1 | 0 | 0 | 4 | | Hypothesis-generating only |
| CYP enzymes | 0 | 2 | 0 | 0 | 0 | 1 | 0 | 3 | | Unsupported |
| LdCS | 0 | 1 | 0 | 0 | 1 | 1 | 0 | 3 | | Hypothesis-generating only; unlikely |
| OCT2 | 0 | 0 | 0 | 0 | 1 | 1 | 0 | 2 | | Hypothesis-generating only |
| CK2 | 0 | 0 | 0 | 0 | 1 | 1 | 0 | 2 | | Hypothesis-generating only |
| PD-L1, CTLA-4, PD-1 | 0 | 0 | 0 | 0 | 1 | 1 | 0 | 2 | | Hypothesis-generating only; unlikely |
| CD80 | 0 | 0 | 0 | 0 | 1 | 1 | 0 | 2 | | Unsupported; unlikely |
| M^pro^ | 0 | 0 | 0 | 0 | 1 | 1 | 0 | 2 | | Hypothesis-generating only |
| LdGlyRS | 0 | 0 | 0 | 0 | 1 | 1 | 0 | 2 | | Hypothesis-generating only |
| Phosphofructokinase | 0 | 0 | 0 | 0 | 1 | 1 | 0 | 2 | | Hypothesis-generating only |
| Pyruvate kinase | 0 | 0 | 0 | 0 | 1 | 1 | 0 | 2 | | Hypothesis-generating only |
| hERG | 0 | 0 | 0 | 0 | 0 | 1 | 0 | 1 | | Hypothesis-generating only; Uncertain clinical relevance / tissue-dependent |
| BACE-1 | 0 | -1 | 0 | 0 | 0 | 1 | 0 | 0 | | Unsupported |

**Supporting Table 2. Docking score values of BZA targets and/or potential targets found in the literature.**

| **Protein** | **Molecular docking software** | **Molecular docking value** (kcal/mol) | **Reference** |
| --- | --- | --- | --- |
| ERα | GRIP Batch Docking | -81.99 | ^46^ |
| ALDH1A1 | BioMolTech’s Lead Finder docking algorithm | -15.44 | ^49^ |
| PD-L1 homodimer | AutoDock Vina | -10.7 | ^50^ |
| ERα | AutoDock Vina | -10.5 | ^48^ |
| ERα | Glide | -9.33 | ^45^ |
| ERα | Glide | -9.48 | ^44^ |
| LdGlyRS | AutoDock Vina | -9.4 | ^51^ |
| Oxidized PDI | Glide | -9.34 to -8.37 | ^55^ |
| Reduced PDI | Glide | -9.63 to -8.91 | ^55^ |
| GP130 | MSLD (multiple fragment simultaneous docking screening and drug repositioning) | -8.2 | ^10^ |
| CK2 | Glide module | -7.937 | ^52^ |
| ERα | Glide | -7.331 | ^47^ |
| WDHD1 | Schrödinger software | -7.173 | ^11^ |
| AOX1 | Plants program | -6.72 | ^53^ |
| PD-1 | AutoDock Vina | -6.6 | ^50^ |
| ACE2/RBD | Autodock Vina  Glide | -6.24 | ^54^ |
| CTLA-4 | AutoDock Vina | -6.2 | ^50^ |
| PD-L1 | AutoDock Vina | -6.1 | ^50^ |
| BACE-1 | Glide module | -6.07 | ^38^ |
| CD80 | AutoDock Vina | -4.7 | ^50^ |

**Supporting Table 3. Gene Chip and RNAseq detailed data of cell lines and tissues treated with BZA alone or in combination with estrogen compounds (E2, CE, EC10 or CEE).**

| **Reference** | **Model** | **Method** | **Results** |
| --- | --- | --- | --- |
| ^42^ | MCF7  Culture media: DMEM/F12 (phenol red free) + 10% FBS + 1X GlutaMAX-1 | Media for gene chip: DMEM/F12 (phenol red free) + 10% DC-FBS + 1X GlutaMAX-1 -> after 24h remove for 2% DC-FBS  GeneChip : Affymetrix Human Genome U133 plus 2.0 Array  BZA concentration: UNK  CE concentration: UNK    Treatment(s) duration: UNK | No information on specific genes  **Standalone treatment** (*BZA, RAL and LAS used alone)*:   - 8 genes were modulated up to 3-fold change (up or downregulation) in BZA and RAL treatment. (p-value < 0.01) - 2 genes were modulated up to 3-fold change (up or downregulation) in BZA and LAS treatment. (p-value < 0.01) - 40 genes were modulated up to 2-fold in BZA and RAL (up or downregulation) treatment. (p-value < 0.01) - 21 genes were modulated up to 2-fold in BZA and LAS (up or downregulation) treatment. (p-value < 0.01)   **Combination treatment (***BZA+CE vs CE)*:   - Similar pattern of expression for 339 genes of four gene sets (up or downregulation). However, these genes were inhibited with LAS and RAL. - BZA modified the expression of 298/1163 CE-regulated genes. However, these genes were more inhibited with LAS and RAL. |
| ^91^ | MCF7  Culture media: DMEM/F12+ 10% FBS + 1X GlutaMAX-1 | Media for gene chip: DMEM/F12 (phenol red free) + 10% DC-FBS + 1X GlutaMAX-1 -> after 24h remove for 2% DC-FBS  Gene chip: Affymetrix Human Genome U133 plus 2.0 Array  BZA concentration: 1 nM or 10 nM  CE or E2 concentration: 1nM  Treatment(s) duration: 24h | **Standalone treatment:**  53 genes modulated with 1 nM and 10 nM BZA alone compared to vehicle (t-test p-value < 0.01; fold change >3):   - Upregulated genes: *HTR4, DUSP1, EGR1, MSI1, FOS, KCNT2, EGR2, CTGF, C9orf71, CYR61, MDFI, ZNF264, LAMB1, ZNF479, RBAF600, CDC42, RNF146, PITRM1, KIF13A, GPR87, FLJ25692, SCAMPER, CDH7, DDEF1, NFKBIZ, ZNF10, ADAMTS5* - Downregulated: *UHRF1, ZNF367, DTL, CDC25A, GREB1, MCM10, FANCB, HELLS, MNAB, SEZ6L2, MGP, C1QTNF6, RPESP, MYBL1, ATRNL1, COL12A1*   Clustering of genes that are ER-responsive: heatmap pattern of BZA alone is similar to vehicle and is different to E2 and CE; it is also similar to RAL.  Clustering of SERMs responsive genes (genes that are unchanged upon E2 or CE treatments, but are upregulated upon SERMs treatments):   - BZA and RAL induce similar pattern of expression - BZA + CE does not cluster with the other combinations (LAS + CE or RAL + CE) - BZA + CE clustered with CE and E2   **Combination treatment**:  CE-modulated genes that are antagonized by BZA (mostly upregulated by CE but downregulated with BZA): 54 genes   - Ex: *FOS, JAK2, PK1B, PRSS23, TOP2A, AREG, NTRK2, ENPP3* - Molecular functions: cancer, cellular growth and proliferation, RNA damage repair, cell cycle - Signaling pathways: cell cycle G2M DNA damage checkpoint regulation, JAK/Stat signaling, ERK/MAPK signaling, EGF signaling   CE-modulated genes that are not antagonized by BZA (mostly downregulated by CE but unchanged with BZA): 76 genes   - EX: *CDKN2B, TGFB2, IL1R1, DRD1, TNFSF10, MYBL1, NT5E* - Molecular functions: cancer, cellular growth and proliferation, cardiovascular system function and development, cell cycle - Signaling pathways: NFκB signaling, cell cycle G1/S checkpoint regulation, p30 MAPK signaling, TGFβ-signaling, WTN/β-catenin signaling |
| ^92^ | MCF7  Culture media: DMEM/F12+ 8% FBS + nonessential amino acids + sodium pyruvate | Media for gene chip: DMEM/F12 (phenol red free) + 8% DC-FBS  Gene chip: Affymetrix Human Genome U133 plus 2.0 Array  BZA concentration: 100nM  E2 concentration: 1nM  Treatment(s) duration: 24h | ANOVA, Holm correction α = 0.05  The transcriptomes of MCF7 treated with BZA or RAL clustered. Both treatments transcriptomes also clustered with vehicle.  The transcriptomes of MCF7 treated with BZA + E2 or RAL + E2 clustered.  **BZA’s roles**  Antagonist:   - BZA alone has a similar pattern compared to ICI, a SERD. - BZA in combination with E2 reduces the expression of gene modulated by E2 without altering basal expression levels when used alone.   - ex: *IL17RB* - Annotation of modulated genes: mostly related to cell cycle.   Inverse agonist:   - BZA alone reduces the expression of genes (or stimulates) below the basal level (vehicle). These genes are also modulated in the same direction in combination with E2.   - Ex: *ASCL1* - Annotation of modulated genes: mostly related to metabolism and cell cycle.   Partial agonist/antagonist:   - BZA does not mimic E2 alone but reverse E2-induced modulation of genes expression when given in combination with E2.   E2-independent:   - BZA modulates genes that are not modulated by E2. (1.5-fold: up or down; Gene selection with RAL) - Genes modulated: *MAOB, SLFN5, SLC2A1, STC2, DNASE1L2, ANXA9, GPR56, THRAP2, SULF1, HLADRB1, HLADRB3, TRAF31P2, FER1L3, SCL24A3, RGNEF, ARRDC4, CTHRC1, PPM1K, FLJ42957* |
| ^93^ | MCF7  Culture media: DMEM/F12 (phenol red free) + 10% FBS | Media for RNAseq: DMEM/F12 (phenol red free) + 2% DC-FBS (48h prior to RNAseq)  RNA-seq: Illumina  BZA concentration: 5nM  E2 or EC10 concentration: 1nM  Treatment(s) duration: 24h | Fold-change >2 and adjusted p-value < 0.05  The transcriptomes of MCF7 treated with BZA or vehicle clustered.  The transcriptomes of MCF7 treated with BZA + E2 or BZA + EC10 clustered.  **Standalone treatment**  BZA alone: only a few genes are modulated (no report of these genes).  **Combination treatment**  BZA + E2 or EC10:   - BZA reduces the expression of genes stimulated by E2 or EC10 -> Biological processes: RNA processing, organic cyclic compound metabolic process, regulation of cell cycle - Genes unchanged by E2 or EC10 alone but reduced upon combination of E2/EC10 with BZA -> biological processes: regulation of transcription by RNA polymerase II, cell surface receptor signaling pathway, signaling   BZA-tempered (ER responsive that BZA alters):   - Ex: *GREB1*, *NRIP1*, *PGR* - Ex: Estrogen-dependent gene expression pathways, SUMOylation of DNA damage and repair proteins, AURKA Activation by TPX2, Amplification of signal from unattached kinetochores via a MAD2 inhibitory signal   BZA modulated genes that are unchanged by E2 or EC10:  BZA-inhibited:   - Ex: *NRAS*, *PAK2*, *PPKACA* - Ex: Estrogen-dependent gene expression pathways, SUMOylation of DNA damage and repair proteins, COPI-mediated anterograde transport, Deactivation of the beta-catenin transactivation complex   BZA-activated:   - Ex: *ANKRD9*, *KLHL21*, *UBE2M* - Rho GTPase and eukaryotic translation termination pathways |
| ^93^ | MCF10A  Culture media: DMEM/F12 (phenol red free) + 5% HS + 10µg/ml human insulin + 20 ng/ml recombinant hEGF +100 ng/ml cholera toxin + 0.5 µg/ml Hydrocortisone+ Pen/Strep + Glutamine | Media for RNAseq: DMEM/F12 (phenol red free) + 2% DC-serum+ 10µg/ml human insulin + 20 ng/ml recombinant hEGF +100 ng/ml cholera toxin + 0.5 µg/ml Hydrocortisone+ Pen/Strep + Glutamine (48h prior to RNAseq)  RNA-seq: Illumina  BZA concentration: 5nM  E2 or EC10 concentration: 1nM  Treatment(s) duration: 24h | Fold-change >2 and adjusted p-value < 0.05  No clustering of RNAseq data between treatments.  Low differential expression among treatments. |
| ^61^ | Liver C57BL/6J OVX high fat diet | RNA-seq: Illumina  BZA concentration: 3mg/kg/day  E2: 5µg/kg/day  CE: 2.5 mg/kg/day  Treatment(s) duration: 6 weeks | Fold-change >2 and FDR (or q) < 0.05  **Standalone treatment and combination treatment**  Upregulated genes by BZA:   - 222 genes not shared with other treatments E2, CE and BZA + CE - 345 genes shared with E2 or CE or BZA + CE   Downregulated genes by BZA:   - 169 genes not shared with other treatments E2, CE and BZA + CE - 288 genes shared with E2 or CE or BZA + CE   Upregulated GO TERMS/pathways (based on BZA + CE but also in BZA and E2 or CE alone): Negative_regulation_of_inflammatory_response, NF-Kappa_B_signaling pathway, Reactive_oxygen_species_pathway, Coagulation  Downregulated GO TERMS/pathways (based on BZA + CE but also in BZA and E2 or CE alone): Fatty_acid_metabolism, Nuclear_receptors_in_lipid_metabolism_and_toxicity, Fatty_acid_beta-oxidation, Reactive_oxygen_species_pathway |
| ^56^ | Liver C57BL/6J OVX | RNA-seq: Illumina  BZA concentration: 10mg/kg/day  CE: 3 mg/kg/day  Treatment(s) duration: 3 weeks | Fold-change at least 1.5-fold relative to placebo  **Standalone treatment**  Genes that are upregulated by BZA only: *Peo1*  Genes that are downregulated by BZA only: *Foxa2*  Similar heatmap pattern between BZA, CE and BZA + CE |
| ^65^ | Sprague-Dawley rats’ ovaries | Gene chip: Rat Genome RAE230 2.0 arrays  BZA concentration: 30 mg/kg/day  Treatment(s) duration: 4-8 days | **Standalone treatment**  Upregulated (1.7-fold, p-value < 0.01): 77 transcripts  Downregulated (1.7-fold, p-value < 0.01): 94 transcripts  Genes that are modulated by at least 3-fold at 4 days (up to 8 days) (P < 0.01):  Upregulated genes:   - *Svs5, Akr1b8, Ass1, Cml, Hpgd, Cyp17a1, Cyp1b1, Insl3, Abhd3*   Downregulated genes:   - *Xpnpep2, Sfrp4, Myom2, Ccl20, Pla2g1b, Atp1b2* |
| ^94^ | OVX Monkey’s breast | Gene chip: Affymetrix GeneChip Rhesus Macaque Genome Array  BZA concentration: 20 mg/kg/day  Treatment duration: 4-8 days  conjugated equine estrogens (CEE): 0.45 mg/kg/day  Treatment duration: 6 months | (ANOVA) and pairwise comparisons at a fold change >2, adjusted Benjamini & Hochberg P < 0.05  The transcriptomes of breast treated with BZA + CEE or BZA alone clustered, but both did not cluster with CEE alone.  **Combination treatment**  Upregulated genes by CEE   - Unchanged by BZA alone compared to vehicle - Downregulated with BZA+CEE treatment compared to vehicle: - Ex: *UQCRC2*   Upregulated genes by BZA+CEE:   - *HLA-DPA1, IGHA1, GZMB, GZMK, CCL5, UBD, MDM4*   **Standalone treatment**  1 gene was upregulated by BZA alone: *PGR* |

Abbreviations: BZA= bazedoxifene, RAL= raloxifene, LAS= lasofoxifene, ICI= fluvestrant. SERD= selective estrogen receptor degrader, SERM= selective estrogen receptor modulator, UNK= unknown, FDR= false discovery rate, E2=estradiol, CE= conjugated estrogens, EC10= estrogen compounds, CEE= conjugated equine estrogens, OVX = ovariectomized.
